# Disseminated intravascular coagulation immediately after trauma predicts a poor prognosis in severely injured patients

**DOI:** 10.1038/s41598-021-90492-0

**Published:** 2021-05-26

**Authors:** Takeshi Wada, Atsushi Shiraishi, Satoshi Gando, Kazuma Yamakawa, Seitaro Fujishima, Daizoh Saitoh, Shigeki Kushimoto, Hiroshi Ogura, Toshikazu Abe, Toshihiko Mayumi, Junichi Sasaki, Joji Kotani, Naoshi Takeyama, Ryosuke Tsuruta, Kiyotsugu Takuma, Norio Yamashita, Shin-ichiro Shiraishi, Hiroto Ikeda, Yasukazu Shiino, Takehiko Tarui, Taka-aki Nakada, Toru Hifumi, Kohji Okamoto, Yuichiro Sakamoto, Akiyoshi Hagiwara, Tomohiko Masuno, Masashi Ueyama, Satoshi Fujimi, Yutaka Umemura, Yasuhiro Otomo

**Affiliations:** 1 Division of Acute and Critical Care Medicine, Department of Anesthesiology and Critical Care Medicine, Hokkaido University Faculty of Medicine, Sapporo, Japan; 2 Emergency and Trauma Center, Kameda Medical Center, Kamogawa, Japan; 3 Department of Acute and Critical Care Medicine, Sapporo Higashi Tokushukai Hospital, Sapporo, Japan; 4 Department of Emergency Medicine, Osaka Medical College, Osaka, Japan; 5 Center for General Medicine Education, Keio University School of Medicine, Tokyo, Japan; 6 Division of Traumatology, Research Institute, National Defense Medical College, Saitama, Japan; 7 Division of Emergency and Critical Care Medicine, Tohoku University Graduate School of Medicine, Sendai, Japan; 8 Department of Traumatology and Acute Critical Medicine, Osaka University Graduate School of Medicine, Osaka, Japan; 9 Department of Emergency and Critical Care Medicine, Tsukuba Memorial Hospital, Tsukuba, Japan; 10 Health Services Research and Development Center, University of Tsukuba, Tsukuba, Japan; 11 Department of Emergency Medicine, School of Medicine, University of Occupational and Environmental Health, Kitakyushu, Japan; 12 Department of Emergency and Critical Care Medicine, Keio University School of Medicine, Tokyo, Japan; 13 Division of Disaster and Emergency Medicine, Department of Surgery Related, Kobe University Graduate School of Medicine, Kobe, Japan; 14 Advanced Critical Care Center, Aichi Medical University Hospital, Nagakute, Japan; 15 Advanced Medical Emergency and Critical Care Center, Yamaguchi University Hospital, Ube, Japan; 16 Emergency and Critical Care Center, Kawasaki Municipal Hospital, Kawasaki, Japan; 17 Department of Emergency and Critical Care Medicine, School of Medicine, Kurume University, Kurume, Japan; 18 Department of Emergency and Critical Care Medicine, Aizu Chuo Hospital, Aizuwakamatsu, Japan; 19 Department of Emergency Medicine, Trauma and Resuscitation Center, Teikyo University School of Medicine, Tokyo, Japan; 20 Department of Acute Medicine, Kawasaki Medical School, Kurashiki, Japan; 21 Department of Trauma and Critical Care Medicine, Kyorin University School of Medicine, Tokyo, Japan; 22 Department of Emergency and Critical Care Medicine, Chiba University Graduate School of Medicine, Chiba, Japan; 23 Department of Emergency and Critical Care Medicine, St. Luke’s International Hospital, Tokyo, Japan; 24 Department of Surgery, Center for Gastroenterology and Liver Disease, Kitakyushu City Yahata Hospital, Kitakyushu, Japan; 25 Emergency and Critical Care Medicine, Saga University Hospital, Saga, Japan; 26 Center Hospital of the National Center for Global Health and Medicine, Tokyo, Japan; 27 Department of Emergency and Critical Care Medicine, Nippon Medical School, Tokyo, Japan; 28 Department of Trauma, Critical Care Medicine, and Burn Center, Japan; Community Healthcare Organization, Chukyo Hospital, Nagoya, Japan; 29 Division of Trauma and Surgical Critical Care, Osaka General Medical Center, Osaka, Japan; 30 Trauma and Acute Critical Care Center, Medical Hospital, Tokyo Medical and Dental University, Tokyo, Japan

**Keywords:** Outcomes research, Trauma

## Abstract

Trauma patients die from massive bleeding due to disseminated intravascular coagulation (DIC) with a fibrinolytic phenotype in the early phase, which transforms to DIC with a thrombotic phenotype in the late phase of trauma, contributing to the development of multiple organ dysfunction syndrome (MODS) and a consequently poor outcome. This is a sub-analysis of a multicenter prospective descriptive cross-sectional study on DIC to evaluate the effect of a DIC diagnosis on the survival probability and predictive performance of DIC scores for massive transfusion, MODS, and hospital death in severely injured trauma patients. A DIC diagnosis on admission was associated with a lower survival probability (Log Rank *P* < 0.001), higher frequency of massive transfusion and MODS and a higher mortality rate than no such diagnosis. The DIC scores at 0 and 3 h significantly predicted massive transfusion, MODS, and hospital death. Markers of thrombin and plasmin generation and fibrinolysis inhibition also showed a good predictive ability for these three items. In conclusion, a DIC diagnosis on admission was associated with a low survival probability. DIC scores obtained immediately after trauma predicted a poor prognosis of severely injured trauma patients.

## Introduction

The two major insults of trauma and sepsis induce systemic inflammatory response syndrome (SIRS), which affects a patient’s outcome due to multiple organ dysfunction syndrome (MODS)^1^. In addition to SIRS, the influence of a compensatory anti-inflammatory response syndrome on the development of organ dysfunction development has been detected^2^. However, all randomized controlled trials targeting SIRS failed, so another hypothesis for improving the outcome of these patients began to be explored at the end of the 1990s^3^.

Tight molecular links between inflammation and coagulation have been described^4^, and crosstalk between innate immune inflammation and coagulation has been considered a leading cause of MODS^5^. Disseminated intravascular coagulation (DIC), defined as dysregulated inflammatory and coagulofibrinolytic responses to insults such as trauma and sepsis, can give rise to MODS via the bidirectional interplay between SIRS and systemic thrombin generation associated with endothelial injury^6^. Thus, DIC is a representative pathological syndrome embodying the close association of inflammation with coagulation.

Cellular injury due to trauma releases mitochondrial damage-associated molecular patterns (DAMPs) such as mitochondrial DNA, which induce SIRS and then elicits neutrophil-mediated organ injury^7^. Histones are major DAMPs released from injured cells or activated neutrophils, forming neutrophil extracellular traps (NETs), which lead to inflammation, activation of coagulation, insufficient anticoagulation controls, impaired fibrinolysis and cytotoxic effects causing tissue damage^8^. All of these changes are characteristics of DIC; therefore, histones and NETs are considered major pathomechanisms of DIC^6,8,9^. Markedly elevated histones immediately after trauma induce proinflammatory cytokine release, coagulation activation, endothelial damage, and NETs formation, which result in DIC and consequent microvascular thrombosis and MODS^10^.

DIC is recognized as thrombohemorrhagic disorder^6^. In clinical settings, two phenotypes of DIC have been reported: fibrinolytic and thrombotic phenotypes^9^. DIC with a fibrinolytic phenotype at an early phase of trauma contributes to a poor prognosis due to massive bleeding^11^. During the late period of trauma, the combined activation of coagulation and inflammation in DIC plays an important role in the development of MODS and consequent poor outcome after severe trauma^12^. Furthermore, DIC and sustained SIRS for more than 3 days after trauma were found to predict MODS with a likelihood ratio of 11.5 and 6.25, respectively^13^. A database analysis of four published scales showed that the ability to detect polytrauma patients at risk of complications in both the early and late phases could be improved by adding different scales related to hemorrhaging and coagulation^14^. This finding suggests the significance of hemorrhaging and thrombosis, namely DIC as a thrombohemorrhagic disorder, for predicting the outcome of trauma patients.

The present study evaluated the utility of the DIC scores for detecting massive transfusion and MODS as well as hospital death as results of these events and investigated the influence of the DIC diagnosis on the survival probability in severely injured trauma patients. The influence of the ability to detect thrombin and plasmin generation and as well as the inhibition of fibrinolysis on these outcomes was also evaluated.

## Methods

### Study design, setting, and ethical approval

This is a prognostic study performed as a sub-analysis of a multicenter prospective descriptive cross-sectional study conducted by the Japanese Association for Acute Medicine (JAAM) Focused Outcomes Research in Emergency Care in Acute Respiratory Distress Syndrome, Sepsis and Trauma (FORECAST) study group^15^. The JAAM FORECAST TRAUMA study recruited participants from April 1, 2016, to January 31, 2018, from 39 emergency departments and intensive-care units (ICUs) in tertiary hospitals and was registered at the University Hospital Medical Information Network Clinical Trial Registry. (UMIN-CTR ID: UMIN000019588). This study was approved under the condition that written informed consent should be obtained from either the patient or their next of kin by the JAAM and the Ethics Committee of each hospital (JAAM, 2014-01; Hokkaido University Graduate School of Medicine, Head institute of the FORECAST group, 014-0307) and was performed in accordance with Declaration of Helsinki.

### Participants

The JAAM FORECAST TRAUMA study enrolled severely injured adult trauma patients (≥ 16 years old) with an Injury Severity Score (ISS) ≥ 16 who were directly transported from the scene by emergency medical services. Patients with a history of cardiac arrest and resuscitation, who were receiving anticoagulants, who had hemorrhagic diathesis or coagulopathy due to any causes, or who had been transferred from other hospitals were excluded before registration. The size of the study population was dependent on the study period. All patients were followed up until discharge.

### Aims and outcomes

The primary aim of the present study was to confirm the utility of a DIC diagnosis and DIC scores immediately after trauma for detecting massive transfusion, MODS, and hospital death of severely injured trauma patients. In addition, the influence of the ability to detect thrombin, plasmin and inhibition of fibrinolysis on the outcomes of trauma patients was investigated. The survival probability and all-cause hospital death were used as primary outcomes. Ventilator-free days and ICU-free days were also obtained as secondary outcomes.

### Definition and the diagnosis

The severity of injury was assessed on an anatomical and physiological basis according to the ISS and revised trauma score, respectively. Severe trauma was defined as an ISS ≥ 16. A DIC diagnosis was made based on the JAAM DIC diagnostic criteria^16^ (Table S1). In the present study, the prothrombin time International Normalized Ratio (INR) was used as a substitute for the prothrombin time ratio for the diagnosis of DIC. Transfusion of packed red blood cells (PRBCs) more than the estimated circulating blood volume (7.5% of body weight) within 24 h after the presentation to the emergency department (ED) met the definition of massive transfusion. Massive transfusion was also assessed by the rate of transfusion, evaluated by the volume of PRBC administered from the presentation at the ED to 3 h after the admission. Organ dysfunction was evaluated using the sequential organ failure assessment (SOFA) score ^17^. To avoid bias arising from overlapping platelet counts in both the SOFA and DIC scores, the original SOFA score and the SOFA score without the coagulation score were calculated. The Glasgow Coma Scale (GCS) for the SOFA score of the central nervous system was used without manipulation. An individual SOFA score ≥ 2 was considered to indicate dysfunction of each organ, and cases of dysfunction in more than two organs without coagulation dysfunction were defined as MODS. The SIRS criteria were used for the assessment of systemic inflammation^1^. A systolic blood pressure < 90 mmHg at the scene or at the ED and lactate levels > 2 mmol/L at the ED were defined as shock. The Charlson index was determined for the assessment of comorbidities^18^. Ventilator-free days was defined as the number of days within the first 28 days after admission during which a patient was able to breathe without a ventilator. ICU-free days were calculated in a similar manner.

### Data collection and measurements

Immediately after arrival at the ED (0-h timepoint) and 3 h after admission (3-h timepoint), 15 mL of blood was collected in citrated tubes at each sampling point. The samples were immediately centrifuged at 4 °C in the laboratories of each hospital, and the obtained plasma was stored at − 80 °C. All plasma samples were measured at the center laboratory of the LSI Medience Corporation (Tokyo Japan). We measured the following molecular markers: (1) soluble fibrin (marker of direct thrombin generation) (LA, IATRO SFII; LSI Medience, Tokyo, Japan), (2) plasmin and α2-plasmin inhibitor (antiplasmin) complex (marker of plasmin generation) (LPIA, LPIA-ACE PPI II; LSI Medience, Tokyo, Japan), (3) plasminogen activator inhibitor-1 (PAI-1) (marker of inhibition of fibrinolysis) (LA, LPIA•tPAI test; LSI Medience, Tokyo, Japan), and (4) D-dimer (marker of fibrinolysis) (LPIA, LPIA GENESIS D-dimer; LSI Medience, Tokyo, Japan). In addition to routine laboratory tests and blood gas analyss, measurements of platelet counts, prothrombin time (sec, INR), activated partial thromboplastin time (APTT), fibrinogen, fibrin/fibrinogen degradation products (FDP), and the FDP/D-dimer ratio were performed at 0-, 3-, and 24-h timepoints after the arrival at the ED. The SIRS criteria and DIC scores were calculated at the 0-, 3-, and 24-h timepoints, and SOFA scores were obtained at 24 h after admission to the ED.

### Statistical methods

Measurements are expressed as the median with the 25th–75th interquartile range or number (percentage). Missing values were used without manipulation. Differences in demographics and measured parameters between two groups (DIC vs. non-DIC) were compared with the Mann–Whitney U-test for continuous variables, and either the chi-square test or Fisher’s exact test was used for nominal variables when required. Time courses of DIC scores, platelet counts, and global markers of coagulation and fibrinolysis (0 h vs. 3 h and 24 h) were also evaluated using the Mann–Whitney U-test. The receiver operating characteristic (ROC) curve was constructed, and the area under the ROC curve (AUC) was used to assess the predictive ability of DIC scores for massive transfusion, MODS, and hospital death. Survival probability curves with and without a DIC diagnosis were derived based on the Kaplan–Meier method. Differences with a two-tailed *P* value of < 0.05 were considered statistically significant. The IBM SPSS 26.0 for MAC OSX software program (IBM Japan, Tokyo, Japan) was used for the statistical analyses and calculations.

### Ethical approval and consent to participate

This study was approved by the JAAM and the Ethics Committee of each hospital waiving written informed consent (JAAM, 2014-01; Hokkaido University Graduate School of Medicine, Head institute of the FORECAST group, 014–0307).

## Results

### Characteristics of the participants

A total of 295 patients were registered. After the assessment of eligibility, 276 enrolled patients were divided into 121 DIC and 155 non-DIC patients based on the data obtained immediately on presentation to the ED (0-h timepoint). The flow diagram showing patient screening and registration is presented as Fig. S1. Demographics of the patients are provided in Table 1. DIC patients had a higher proportion of women than non-DIC patients. Almost all DIC patients (110/121, 90.9%) met the definition of SIRS. Higher ISS and revised trauma scores, and a higher prevalence of shock were observed in DIC patients than in non-DIC patients. The abbreviated injury scale (AIS) for individual organs and the GCS at 0, 3, and 24 h are shown in Table S2. No marked differences in the AIS values for the head were noted, but DIC patients had a lower GCS than non-DIC patients at presentation to the ED, which markedly worsened at 3 and 24 h after presentation. The serial changes in DIC scores, platelet counts, and global markers of coagulation and fibrinolysis are shown in Table S3. DIC patients showed lower platelet counts, a prolonged prothrombin time and APTT, lower fibrinogen levels, and higher FDP levels and FDP/D-dimer ratios than non-DIC patients at 0, 3, and 24 h after presentation.

**Table 1:** Demographics of the patients on admission to the emergency department, volume of transfusion and outcomes.

	Non-DIC	DIC	*P* value
Number of the patients	155	121	
Age (years)	59 (41–69)	56 (42–78)	0.386
Male gender n (%)	111 (71.6)	72 (59.5)	0.040
Charlson comorbidity index	0 (0–0)	0 (0–1)	0.050
Blunt injury n (%)	150 (96.8)	118 (97.5)	0.856
Isolated brain injury n (%)	25 (16.1)	9 (7.4)	0.041
DIC score	3 (1–3)	4 (4–5)	< 0.001
Injury Severity Score	24 (18–29)	29 (24–41)	< 0.001
Revised trauma score	7.8 (6.9–7.8)	6.9 (5.1–7.8)	< 0.001
SIRS criteria	2 (1–2)	3 (3–3)	< 0.001
Total SOFA score 24 h after admission	4 (2–7)	7 (4–9)	< 0.001
Central nervous system	0 (0–2)	3 (1–4)	< 0.001
Cardiovascular	0 (0–1)	1 (0–1)	0.008
Respiration	1 (0–2)	1 (1–2)	0.034
Coagulation	0 (0–1)	1 (0–2)	< 0.001
Liver	0 (0–1)	0 (0–1)	0.031
Renal	0 (0–0)	0 (0–0)	0.001
SOFA score without coagulation	3 (2–6)	5 (3–7)	< 0.001
Shock n (%)	23 (14.8)	38 (31.7)	0.001
Lactate (mmol/L)	2.3 (1.6–3.4)	2.9 (2.0–5.0)	0.002
Body temperature (^o^C)	36.6 (36.1–36.9)	36.2 (35.6–36.7)	0.002
Tranexamic acid n (%)	69 (44.5)	66 (54.5)	0.115
Operation within 24 h after admission n (%)	77 (51.0)	73 (61.9)	0.084
Time from injury to ED	45 (35–64)	49 (32–76)	0.571
Intravenous fluids prior to ED (ml)	0 (0–100)	0 (0–200)	0.060
3-h transfusion			
Packed red blood cells (ml)	0 (0–0)	0 (0–840)	< 0.001
Fresh frozen plasma (ml)	0 (0–0)	0 (0–480)	< 0.001
Platelet concentrate (U)	0 (0–0)	0 (0–0)	0.033
24-htransfusion			
Packed red blood cells (ml)	0 (0–280)	1120 (0–3360)	< 0.001
Fresh frozen plasma (ml)	0 (0–240)	960 (0–2400)	< 0.001
Platelet concentrate (U)	0 (0–0)	0 (0–10)	< 0.001
Outcomes			
Massive transfusion n (%)	8 (5.3)	22 (18.6)	< 0.001
MODS 24 h after admission n (%)	28 (42.4)	38 (57.6)	0.021
Organ dysfunction 24 h after admission	1 (0–2)	1 (1–2)	< 0.001
Ventilator-free days	28 (23–28)	21 (0–28)	< 0.001
ICU-free days	24 (18–26)	20 (10–25)	0.001
Hospital death n (%)	5 (3.2)	24 (20.2)	< 0.001

Values are expressed as the median with the 25th–75th interquartile range or number (percentage).

*DIC* disseminated intravascular coagulation, *ED* emergency department, *SIRS* systemic inflammatory response syndrome, *SOFA* sequential organ dysfunction assessment, *ICU* intensive care unit, *MODS* multiple organ dysfunction syndrome. Missing data of Outcomes from upper to bottom, 5, 95, 95, 8, 35, 3, respectively.

### Outcomes of the participants

DIC patients were transfused with larger volumes of platelet concentrate, fresh-frozen plasma (FFP), and PRBCs at 3 and 24 h after admission to the ED than non-DIC patients (Table 1). As a result, the frequency of massive transfusion in DIC patients was higher than that in non-DIC patients (*P* < 0.001) (Table 2). The total SOFA scores, SOFA scores without coagulation scores, and all individual organ SOFA scores were significantly higher in DIC patients than in non-DIC patients. DIC patients showed a higher incidence of MODS than non-DIC patients (*P* = 0.021) and DIC patients showed more organs with dysfunction than non-DIC patients (*P* < 0.001). The mortality rate was higher (*P* < 0.001) and the duration of both ventilator use and ICU stay was shorter in the DIC patients than in the non-DIC patients. No patients died within 3 h after admission. All 9 patients who died within 24 h had a DIC diagnosis, and their DIC score deteriorated from 0 to 3 h. The frequencies of shock and massive transfusion in these 9 patients at 3 h were 55.6% and 33.3%, respectively (Table S4).

**Table 2:** Outcomes of the patients.

	Non-DIC	DIC	*P* value
Massive transfusion n (%)	8 (5.3)	22 (18.6)	< 0.001
MODS 24 h after admission n (%)	28 (42.4)	38 (57.6)	0.021
Organ dysfunction 24 h after admission	1 (0–2)	1 (1–2)	< 0.001
Ventilator-free days	28 (23–28)	21 (0–28)	< 0.001
ICU-free days	24 (18–26)	20 (10–25)	0.001
Hospital death n (%)	5 (3.2)	24 (20.2)	< 0.001

Values are expressed as the median with the 25th–75th interquartile range or number (percentage).

*DIC* disseminated intravascular coagulation, *ICU* intensive care unit, *MODS* multiple organ dysfunction syndrome. Missing data from upper to bottom, 5, 95, 95, 8, 35, 3, respectively.

### Survival probability and outcome prediction

**Figure 1:**
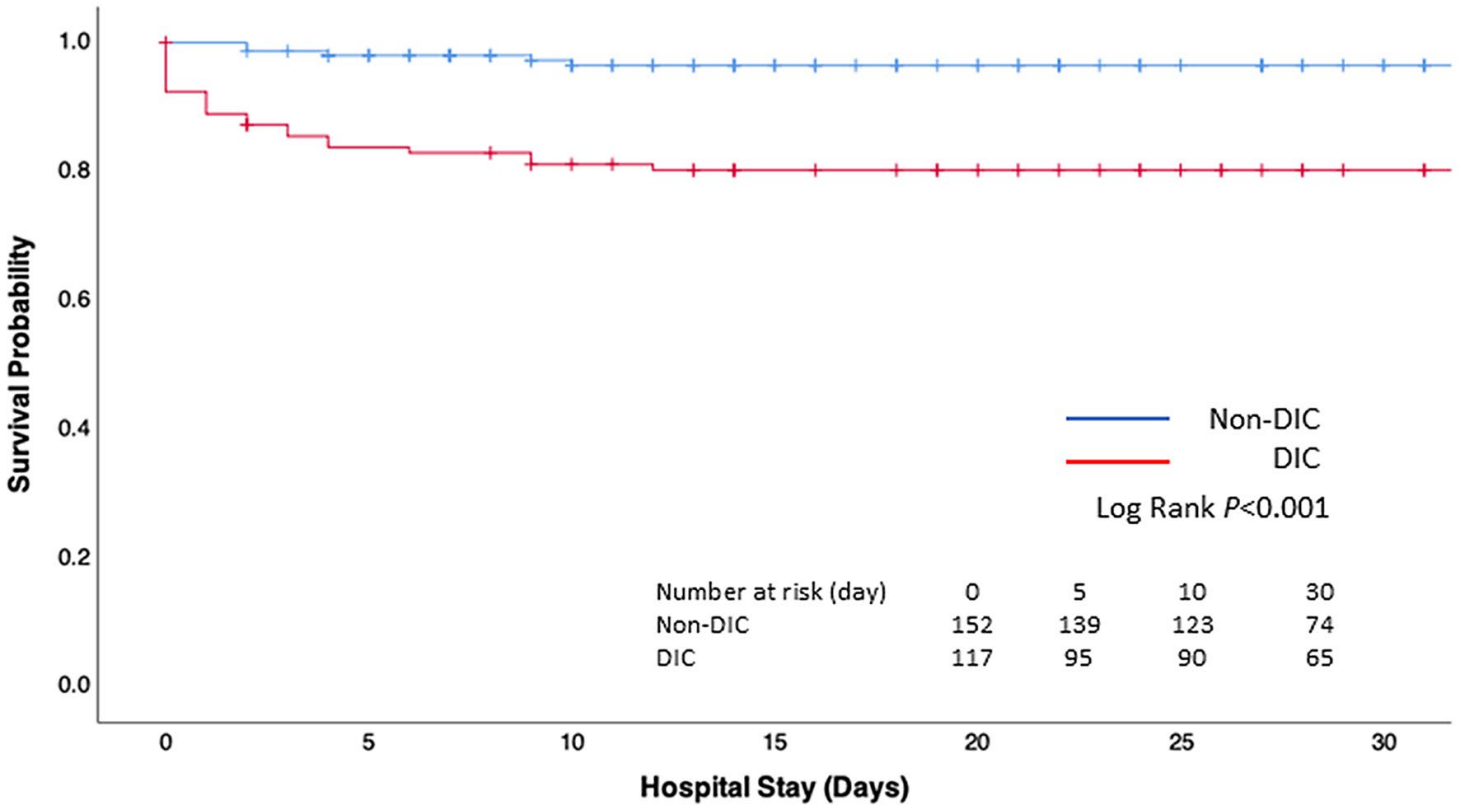
Kaplan–Meier survival probability curves for mortality during hospitalization. Numbers at risk represent the number of patients with or without disseminated intravascular coagulation (DIC) at risk of death at the indicated days.

**Figure 2:**
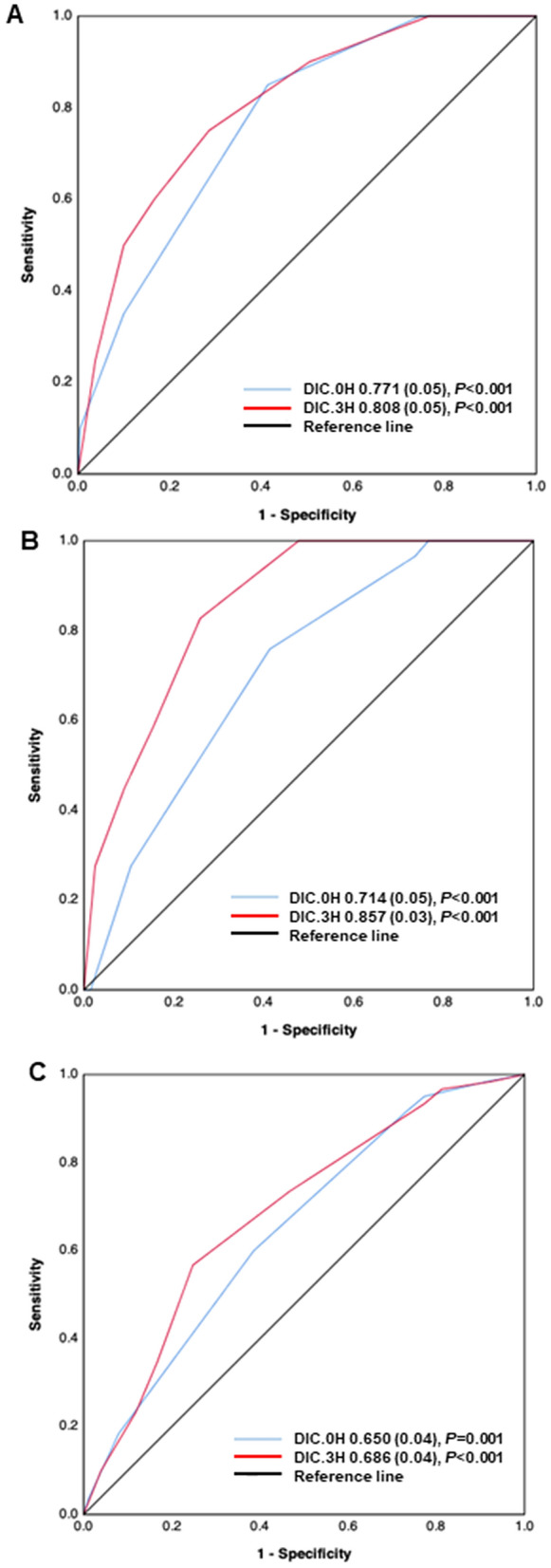
Receiver operating characteristic (ROC) curves of disseminated intravascular coagulation (DIC) scores at 0 and 3 h after presentation to the emergency department (ED) to predict hospital death (**A**), massive transfusion (**B**), and multiple organ dysfunction syndrome (MODS) (**C**) at 24 h after presentation to the ED. The number indicates the area under the ROC curve (AUC) (standard error).

The survival probability of the patients diagnosed with DIC immediately after presentation to the ED was significantly lower than that in those without DIC (Log Rank *P* < 0.001) (Fig. 1). As shown in Fig. 2, the DIC scores immediately after presentation to the ED (0-h timepoint) and at 3 h after admission (3-h timepoint) showed a significant predictive ability for hospital death (Fig. 2A), massive transfusion (Fig. 2B) and the development of MODS (Fig. 2C) at 24 h after admission.

**Figure 3:**
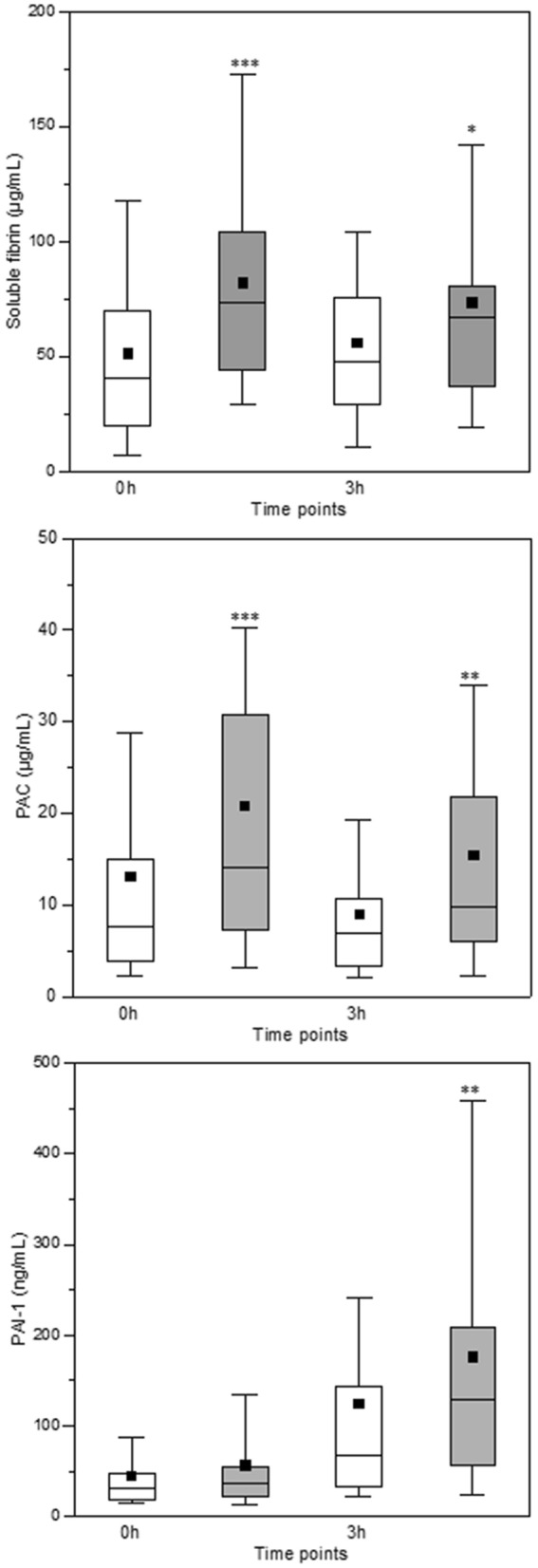
Box plots for soluble fibrin, plasmin and antiplasmin complex (PAC), and plasminogen activator inhibitor-1 (PAI-1) at 0 and 3 h after presentation to the emergency department. Disseminated intravascular coagulation (DIC) patients showed significantly higher values of soluble fibrin and PAC at 0 and 3 h and PAI-1 at 3 h than did non-DIC patients. White box, non-DIC patients; grey box, DIC patients. Horizontal bars in the box indicate the median (middle) and interquartile ranges (upper 25% and lower 75%). Black boxes are mean values. *P < 0.05, **P < 0.01, ***P < 0.001 versus non-DIC patients.

**Figure 4:**
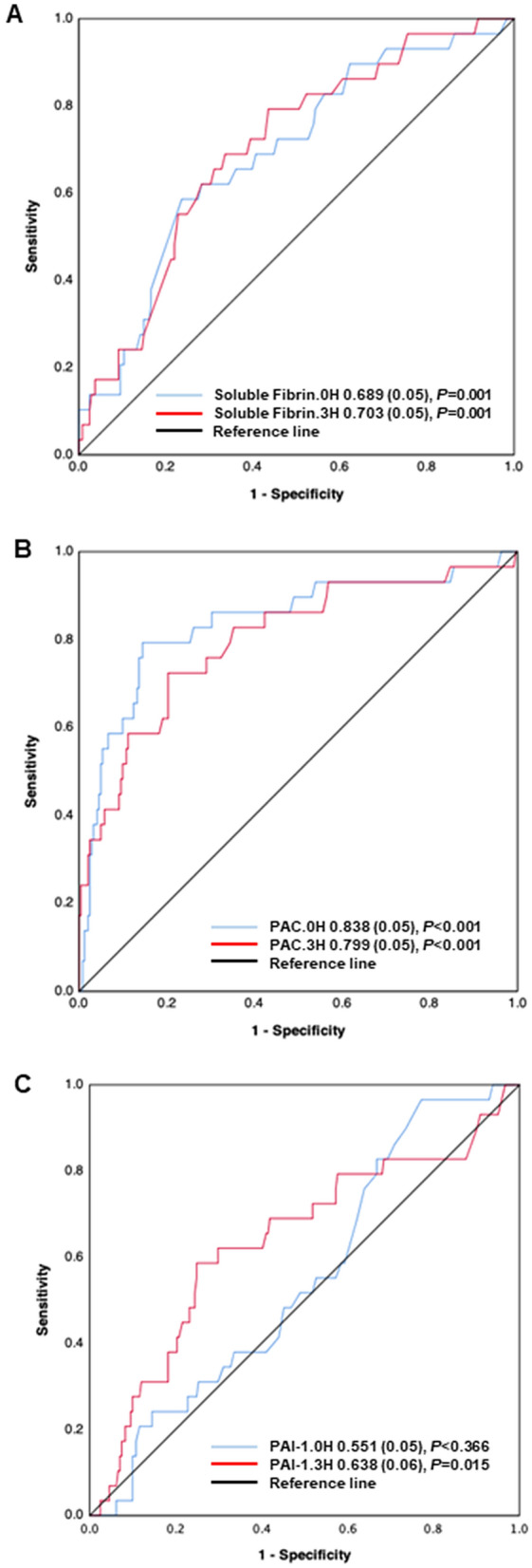
Receiver operating characteristics (ROC) curves of soluble fibrin, plasmin and antiplasmin complex (PAC) and plasminogen activator inhibitor-1 (PAI-1) at 0 and 3 h after presentation to the emergency department to predict hospital death. The number indicates the area under the ROC curve (AUC) (standard error).

The levels of soluble fibrin and plasmin antiplasmin complex at the 0- and 3-h timepoints and PAI-1 at the 3-h timepoint are shown as box plots in Fig. 3. The values were higher in DIC patients than in non-DIC patients. The AUCs of soluble fibrin (*P* = 0.001) and plasmin antiplasmin complex (*p* < 0.001) at the 0- and 3-h timepoints and PAI-1 (*P* = 0.015) at the 3-h timepoint for predicting hospital death were all significant (Fig. 4). In addition, the levels of soluble fibrin at the 0-h timepoint, plasmin antiplasmin complex at the 0-h timepoint, and PAI-1 at the 3-h timepoint showed a significant predictive performance for massive transfusion and MODS at 24 h after admission (Table S5).

## Discussion

The novel findings of this sub-analysis of a multicenter prospective study are that a diagnosis of DIC immediately after trauma can predict the need for massive transfusion at an early phase of trauma, as well as the development of late-phase MODS and a poor outcome with high accuracy. In addition, the massive generation of thrombin and plasmin followed by the inhibition of fibrinolysis by PAI-1 in DIC patients also showed marked predictivity for a poor prognosis.

DIC immediately after trauma represents a fibrinolytic phenotype^6,9^. In this phenotype of DIC, trauma patients need massive transfusion due to trauma-related bleeding as well as DIC-induced oozing-type bleeding at surgical-site wounds, mucosal lesions, serosal surfaces, and the sites of indwelling catheters, among other points. Massive thrombin formation-induced consumption coagulopathy and the time difference between immediate plasmin generation due to tissue-type plasminogen activator (t-PA) release from the Weibel-Palade bodies and the expression of PAI-1 mRNA leading to plasmin-mediated fibrin(ogen)olysis are main causes of bleeding immediately after trauma^6,9^. Markedly high levels of soluble fibrin in DIC patients, as well as a reduction in platelet counts, low levels of fibrinogen, and prolonged prothrombin time and APTT, foster consumption coagulopathy. Similar findings were repeatedly confirmed in previous studies^15,19,20^. Increased thrombin generation due to the coagulation activation after trauma accounts for decreases in various coagulation factors in acute consumption^21^. Even if the coagulation factors are depleted consumptively, increased coagulation (increases in soluble fibrin) continues due to circulating procoagulants, such as microparticles and the impairment of anticoagulation pathways, including tissue factor pathway inhibitor, thrombomodulin and antithrombin, caused by endothelial injury induced by systemic inflammation after trauma^21^. Low fibrinogen levels as well as elevated FDP levels with high FDP/D-dimer ratios in DIC patients suggest fibrin(ogen)olysis. Immediate increases in the plasmin levels followed by an increase in the PAI-1 levels at 3-h timepoints indirectly support the notion of a time difference between the immediate t-PA release and the PAI-1 mRNA expression. The administration of a median 0 mL of intravenous fluids prior to ED presentation and significant decreases in platelet counts and fibrinogen levels from the 0- to 3-h timepoints despite a large volume of platelet concentrate and FFP transfusion in DIC patients deny dilution coagulopathy.

The robustness of the present study is based on the confirmation of the results of previous studies using two prospectively obtained data points immediately after trauma. A single-center retrospective study showed that DIC with a fibrinolytic phenotype diagnosed within 4 h after arrival at the ED contributed to a poor prognosis due to massive transfusion^11^. A multicenter retrospective study concluded that DIC diagnosed immediately after presentation to the ED could be used to predict massive transfusion for 24 h after admission, with an association between DIC and massive transfusion within 24 h also observed (odds ratio, 4.607, *P* = 0.001)^22^. The Kaplan–Meier curves for the time from presentation to the ED to reaching critical thresholds of routine parameters for massive bleeding showed that the platelet counts, prothrombin time, and fibrinogen levels in DIC patients reached their critical level significantly earlier than those in non-DIC patients^23^. In addition, the time taken from the arrival at the ED to meet the definition of massive transfusion was shorter in DIC patients than in non-DIC patients. Hayakawa et al.^20^ showed that DIC at an early phase of trauma associated with consumption coagulopathy and increased fibrinolysis required more blood transfusions than was noted in non-DIC patients. Given these previous findings, the present results showing a good performance of DIC scores immediately after trauma to predict massive transfusion seem very reasonable.

The key point of DIC after trauma is that thrombin generation always underlies the changes in fibrinolytic systems as shown in Fig. 4^6,9,24^. Regardless of the phenotype, DIC is always associated with massive thrombin generation; DIC with a fibrinolytic phenotype progresses to thrombotic phenotype along with the elevation of PAI-1 levels^20,25^. The activated protein C (APC) hypothesis, which is the other main pathology of trauma-induced coagulopathy^21^, insists on the APC-mediated suppression of coagulation, followed by reduced thrombin generation^26,27^. However, the International Society on Thrombosis & Haemostasis recently stated, “APC can be a possible mechanism of trauma-induced coagulopathy; however, following tissue injury, thrombin generation occurs, which is consistent with other types of coagulopathy and DIC.”^28^. The dynamics of soluble fibrin, plasmin and antiplasmin complex and PAI-1 observed in the present study suggest that this progression occurs approximately 3 h after trauma. Despite equal severity of brain injury (as assessed by the head AIS) between the patients with and without DIC, the aggravation of the GCS during the 24-h time period in DIC patients may suggest DIC-induced secondary brain injury due to the progression of intracranial hemorrhaging or microvascular thrombosis.

The present study is significant for its confirmation that a DIC diagnosis affected the survival probability of severely injured trauma patients. In the present study, DIC scores were obtained immediately after trauma (median 49 min from injury), which can be generalized to other institutions, as the DIC scoring system uses laboratory assays that are commonly available at all hospitals^29^. This rapid DIC scoring system, which can identify changes in the innate immune responses to trauma from physiological to pathological, unlike other simple scoring system such as ABC score^30^, were proven to predict not only the rapid delivery of massive transfusion at an early phase of trauma but also late-phase MODS and a poor outcome. MODS with a high mortality rate is characteristic of DIC^6,8^. Rapid progression of DIC from the fibrinolytic to the thrombotic phenotype by 3 h after arrival to the ED may be the reason for the development of MODS within 24 h after admission. Cabrera et al.^31^ showed that trauma patients complicated with MODS had a specific gene expression within 120 min from injury, implicating the participation of innate immune cells, such as neutrophils at this stage of trauma. In addition, previous studies showing immediate changes in mitochondrial DAMPs, histones, neutrophils, and coagulation after trauma may support the results of the present study highlighting the predictive ability of rapid-onset DIC for the later prognosis of patients^7,8,10^.

Another novel finding of the present study was that soluble fibrin, plasmin and antiplasmin complex, and PAI-1 were able to predict massive transfusion, MODS, and hospital mortality of severe trauma patients. DIC patients were associated with significantly higher levels of soluble fibrin, plasmin and antiplasmin complex and PAI-1 than non-DIC patients. In the main study of our series, increased levels of soluble thrombomodulin, a marker of endothelial injury, were observed in DIC patients^15^. Previous studies showed that MODS was more likely when markers of endotheliopathy, e.g. soluble thrombomodulin, were increased immediately after trauma^32^. The generation of thrombin and plasmin, inhibition of fibrinolysis by PAI-1, and endothelial injury are the main pathomechanisms of DIC^6,8^, supporting the significant utility of DIC for predicting a poor outcome of trauma patients in the present study.

Trauma-induced coagulopathy is defined as a pre-stage of DIC and progresses to DIC as a result of dysregulated inflammatory and coagulofibrinolytic responses to trauma^33^. When the trauma is sufficiently severe, therefore, DIC develops immediately after trauma without passing through the trauma-induced coagulopathy stage^15^. Previous studies have shown that trauma-induced coagulopathy was a significant risk factor of MODS^34^ and an independent predictor of MODS and mortality in cases of severe trauma^35,36^. These studies indirectly strengthen the present finding of the significant performance of DIC in predicting MODS and hospital mortality.

The present study demonstrated that DIC patients had a higher proportion of women than non-DIC patients. Previous studies have suggested that the hypercoagulability of women after injury, which may be attributed to the effects of estrogen, may result in improvements in hemostasis after traumatic hemorrhaging^37–39^. However, another study showed that women were less likely to develop severe trauma-induced coagulopathy than men, while women who developed severe coagulopathy were associated with a more than two-fold increased risk of mortality compared with men who developed severe coagulopathy^40^. These findings suggest that the hypercoagulability of women can protect them from death due to hemorrhaging after trauma, and if the trauma is sufficiently severe, hypercoagulability may change from a physiological state to a pathological state, leading to the development of DIC and a subsequently poor outcome. Since the present study targeted patients with relatively severe trauma of ISS > 16, the proportion of women among DIC patients may have been high.

Several limitations associated with the present study warrant mention. Although this study was a sub-analysis of prospectively collected data, the data were retrospectively analyzed and included missing values. The use of tranexamic acid was equally distributed between the two groups, but the effects of tranexamic acid on the obtained results were not elucidated. The present study was a single national study conducted in a developed country, which may limit the global generalization of the obtained results.

## Conclusions

The present study showed that the diagnosis of DIC immediately after trauma was associated with a lower survival probability and higher mortality rate than no such diagnosis. DIC scores obtained immediately after presentation to the ED were able to predict massive transfusion, MODS, and hospital death of severe trauma patients. Substantial thrombin and plasmin generation followed by elevation of the PAI-1 levels, the main pathomechanisms of DIC, also predicted massive transfusion, MODS, and hospital mortality. Therefore, the recognition of DIC at a very early phase of trauma is important in order to predict the outcome of severely injured trauma patients.

## Supplementary Information

Supplementary Information.

Supplementary Legends.

## Data Availability

The dataset used and/or analyzed during current study are available from the corresponding author on reasonable request.
