# Supplementary material for: Disseminated intravascular coagulation immediately after trauma predicts a poor prognosis in severely injured patients
Source: Sci Rep. 2021 May 26;11:11031. doi: 10.1038/s41598-021-90492-0 (PMC8154895; doi:10.1038/s41598-021-90492-0)
Supplement: Supplementary file 1 — Supplementary Information. [file 41598_2021_90492_MOESM1_ESM.pdf]

Disseminated intravascular coagulation immediately after trauma predicts a poor prognosis in severely injured patients

Takeshi Wada[1]*, Atsushi Shiraishi[2], Satoshi Gando[1,3], Kazuma Yamakawa[4], Seitaro Fujishima[5], Daizoh Saitoh[6], Shigeki Kushimoto[7], Hiroshi Ogura[8], Toshikazu Abe[9,10], Toshihiko Mayumi[11], Junichi Sasaki[12], Joji Kotani[13], Naoshi Takeyama[14], Ryosuke Tsuruta[15], Kiyotsugu Takuma[16], Norio Yamashita[17], Shin-ichiro Shiraishi[18], Hiroto Ikeda[19], Yasukazu Shiino[20], Takehiko Tarui[21], Taka-aki Nakada[22], Toru Hifumi[23], Kohji Okamoto[24], Yuichiro Sakamoto[25], Akiyoshi Hagiwara[26], Tomohiko Masuno[27], Masashi Ueyama[28], Satoshi Fujimi[29], Yutaka Umemura[8], Yasuhiro Otomo[30]

[1]Division of Acute and Critical Care Medicine, Department of Anesthesiology and Critical Care Medicine, Hokkaido University Faculty of Medicine, Japan

[2]Emergency and Trauma Center, Kameda Medical Center, Japan

[3]Department of Acute and Critical Care Medicine, Sapporo Higashi Tokushukai Hospital, Japan

[4]Department of Emergency Medicine, Osaka Medical College, Japan

[5]Center for General Medicine Education, Keio University School of Medicine, Japan

[6]Division of Traumatology, Research Institute, National Defense Medical College, Japan

[7]Division of Emergency and Critical Care Medicine, Tohoku University Graduate School of Medicine, Japan

[8]Department of Traumatology and Acute Critical Medicine, Osaka University Graduate School

of Medicine, Japan

[9]Department of Emergency and Critical Care Medicine, Tsukuba Memorial Hospital, Tsukuba, Japan

[10]Health Services Research and Development Center, University of Tsukuba, Japan

[11]Department of Emergency Medicine, School of Medicine, University of Occupational and Environmental Health, Japan

[12]Department of Emergency and Critical Care Medicine, Keio University School of Medicine, Japan

[13]Division of Disaster and Emergency Medicine, Department of Surgery Related, Kobe University Graduate School of Medicine, Japan

[14]Advanced Critical Care Center, Aichi Medical University Hospital, Japan

[15]Advanced Medical Emergency & Critical Care Center, Yamaguchi University Hospital, Japan

[16]Emergency & Critical Care Center, Kawasaki Municipal Hospital, Japan

[17]Department of Emergency & Critical Care Medicine, School of Medicine, Kurume University, Japan

[18]Department of Emergency and Critical Care Medicine, Aizu Chuo Hospital, Japan

[19]Department of Emergency Medicine, Trauma and Resuscitation Center, Teikyo University School of Medicine, Japan

[20]Department of Acute Medicine, Kawasaki Medical School, Japan

[21]Department of Trauma and Critical Care Medicine, Kyorin University School of Medicine, Japan

[22]Department of Emergency and Critical Care Medicine, Chiba University Graduate School of Medicine, Japan

[23]Department of Emergency and Critical Care Medicine, St. Luke's International Hospital, Japan

[24]Department of Surgery, Center for Gastroenterology and Liver Disease, Kitakyushu City Yahata Hospital, Japan

[25]Emergency and Critical Care Medicine, Saga University Hospital, Japan

[26]Center Hospital of the National Center for Global Health and Medicine, Japan

[27]Department of Emergency and Critical Care Medicine, Nippon Medical School, Japan

[28]Department of Trauma, Critical Care Medicine, and Burn Center, Japan; Community Healthcare Organization, Chukyo Hospital, Japan

[29]Division of Trauma and Surgical Critical Care, Osaka General Medical Center, Japan

[30]Trauma and Acute Critical Care Center, Medical Hospital, Tokyo Medical and Dental University, Japan

Correspondence and requests for materials should be addressed to Takeshi Wada
Email: twada1@med.hokudai.ac.jp
Division of Acute and Critical Care Medicine, Department of Anesthesiology and Critical Care Medicine, Hokkaido university Graduate School of Medicine
N15, W7, Kita-ku, Sapporo Japan.

Table S1. Japanese Association for Acute Medicine disseminated intravascular coagulation scoring system

		Score
Systemic inflammatory response syndrome criteria		
	≥3	1
	0-2	0
Platelet counts (10^9/L)		
	<80 or a ≥ 50% decrease within 24 h	3
	≥80 to <120 or a≥ 30% decrease within 24 h	1
	≥120	
0		
Prothrombin time (value of patient/normal value)		
	≥1.2	1
	<1.2	0
Fibrin/fibrinogen degradation products (mg/L)		
	≥25	3
	≥10 to <25	1
	<10	0
Diagnosis		
Four points or more		DIC

The detailed scoring system is described in reference 16.

Table S2. Abbreviated injury scale and Glasgow Coma Scale of the patients

	Non-DIC	DIC	*P*-Value
Abbreviated injury scale			
Head	3 (0-4)	3 (0-4)	0.138
Face	0 (0 -1)	0 (0-0)	0.391
Neck	0 (0-0)	0(0-0)	0.597
Thorax	3 (0-4)	3(0-4)	0.007
Abdomen	0 (0-0)	0 (0-2)	0.005
Spine			
Cervical	0 (0-0)	0 (0-0)	0.421
Thoracic	0 (0-0)	0 (0-0)	0.522
Lumbar	0 (0-0)	0 (0-1)	0.001
Upper extremity	0 (0-1)	0 (0-2)	0.169
Lower extremity	0 (0-2)	2 (0-3)	<0.001
External	0 (0-1)	0 (0-1)	0.053
Glasgow Coma Scale			
0 h	14 (11-15)	12 (6-14)	<0.001
3 h	14 (7-15)	7 (3-14)	<0.001
24 h	15 (10-15)	9 (3-14)	<0.001

Values are expressed as the median with the 25th -75th interquartile range.

DIC, disseminated intravascular coagulation

Table S3. Disseminated intravascular coagulation scores, platelet counts, global markers of coagulation and fibrinolysis

	0 h	3 h	24 h
DIC score			
Non-DIC	3(1-3)	3(1-4)	2(1-4)
DIC	4(4-5)c	5(4-6)c,y	4(3-6)c
Platelet counts (10^9/L)			
Non-DIC	220(186-250)	181(130-214)	159(118-194)
DIC	201(161-257)a	132(90-177)c,z	110(79-150)c,z
Prothrombin time (sec)			
Non-DIC	12.1(11.4-13.0)	12.8(12.0-14.0)	12.8(12.2-13.8)
DIC	13.4(12.2-15.0)c	14.5(13.0-15.9)c,y	13.5(12.7-14.6)b
Prothrombin time INR			
Non-DIC	1.02(1.02-1.09)	1.09(1.00-1.15)	1.09(1.03-1.15)
DIC	1.12(1.02-1.23)c	1.20(1.07-1.32)c,y	1.12(1.05-1.22)c
APTT (sec)			
Non-DIC	26.8(24.6-29.8)	29.1(26.4-32.3)	31.3(28.8-34.0)
DIC	29.1(25.8-37.0)c	33.4(27.5-40.6)c,y	33.1(29.0-37.1)z
Fibrinogen (g/L)			
Non-DIC	2.38(2.11-2.85)	2.05(1.70-2.60)	3.02(2.59-3.85)
DIC	2.07(1.61-2.47)c	1.67(1.19-2.08)c,z	2.78(2.38-3.47)a,z
FDP (mg/L)			
Non-DIC	31.0(14.5-87.5)	40.0(13.3-103.8)	17.0(7.0-38.4)
DIC	117.0(60.6-258.5)c	100.5(55.3-198.4)c	32.3(15.3-64.6)c,z
FDP/D-dimer ratio			
Non-DIC	2.21(1.53-3.13)	2.02(1.52-2.58)	2.19(1.87-2.55)
DIC	2.76(2.08-3.52)b	2.31(1.67-3.45)a	2.11(1.80-2.37)z

Values are expressed as the median with the 25th -75th interquartile range.

APTT, activated partial thromboplastin time; DIC, disseminated intravascular coagulation; FDP, fibrin/fibrinogen degradation products; INR, international normalized ratio. a, *P*<0.05; b, *P*<0.01; c, *P*<0.001 vs. non-DIC. x, *P*<0.05; y, *P*<0.01; z, *P*<0.001 vs. 0 h.

Table S4. Demographics of the 9 patients who died within 24-h after arrival

Age (year)	60 (35-88)
Male gender n (%)	7 (77.8)
DIC n (%)	9 (100)
DIC score at 0 h	4 (4-5)
DIC score at 3 h	7 (4-8)
Injury Severity Score	34 (24-52)
Abbreviated injury scale	
Head	5.0 (2.5-5.0)
Shock n (%)	5 (55.6)
Hemodynamics	
0 h	
Systolic blood pressure (mmHg)	71 (52-113)
Diastolic blood pressure (mmHg)	37 (25-59)
Heart rate (beats/min)	104 (93-129)
Respiratory rate (times/min)	15 (11-30)
3 h	
Systolic blood pressure (mmHg)	86 (51-100)
Diastolic blood pressure (mmHg)	45 (24-64)
Heart rate (beats/min)	96 (72-107)
Respiratory rate (times/min)	19 (24)
Massive transfusion n (%)	3 (33.3)
3-h transfusion	
Packed red blood cell (ml)	1400 (280-2100)
Fresh frozen plasma	1440 (240-1680)
Platelet concentrate (U)	0 (0-20)

Values are expressed as the median with the 25th -75th interquartile range or number (percentage).

DIC, disseminated intravascular coagulation

Table S5. Utility of soluble fibrin, plasmin antiplasmin complex, plasminogen activator inhibitor-1 for the detection of massive transfusion and multiple organ dysfunction syndrome within 24 h of and at 24 h after admission

	AUC (SE)	*P*-Value
Massive transfusion		
Soluble fibrin 0 h	0.627 (0.05)	0.023
Soluble fibrin 3 h	0.530 (0.05)	0.588
Plasmin antiplasmin complex 0 h	0.685 (0.05)	0.001
Plasmin antiplasmin complex 3 h	0.552 (0.05)	0.357
Plasminogen activator inhibitor-1 0 h	0.535 (0.05)	0.530
Plasminogen activator inhibitor-1 3 h	0.638 (0.05)	0.014
Multiple organ dysfunction syndrome		
Soluble fibrin 0 h	0.696 (0.04)	<0.001
Soluble fibrin 3 h	0.600 (0.04)	0.025
Plasmin antiplasmin complex 0 h	0.653 (0.04)	0.001
Plasmin antiplasmin complex 3 h	0.640 (0.05)	0.002
Plasminogen activator inhibitor-1 0 h	0.566 (0.05)	0.139
Plasminogen activator inhibitor-1 3 h	0.622 (0.04)	0.006

AUC, area under receiver operating characteristic curve; SE, standard error.

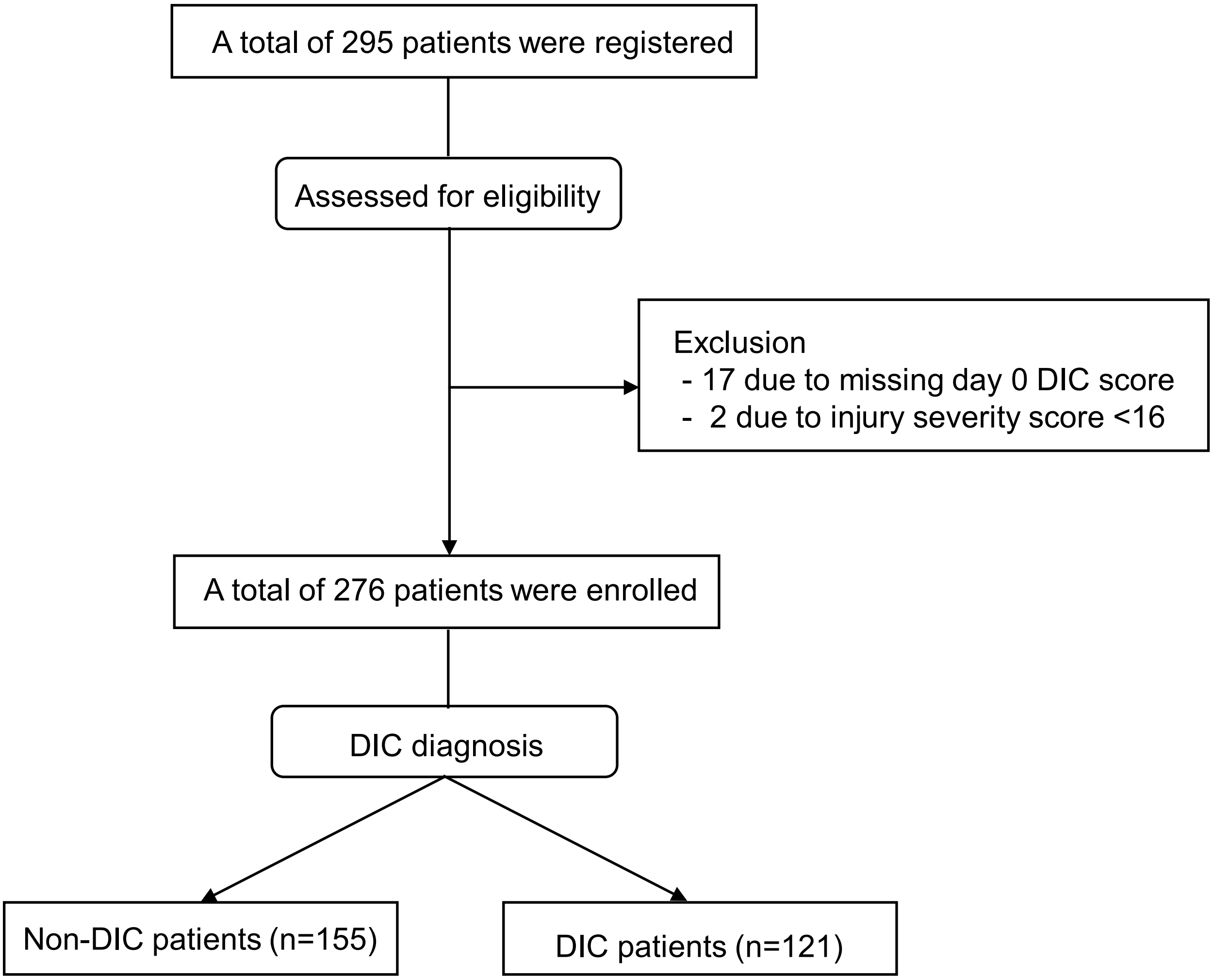
A total of 295 patients were registered
Assessed for eligibility
Exclusion
- 17 due to missing day 0 DIC score
- 2 due to injury severity score <16
A total of 276 patients were enrolled
DIC diagnosis
Non-DIC patients (n=155)
DIC patients (n=121)

Figure S1
