# Supplementary material for: Disseminated intravascular coagulation immediately after trauma predicts a poor prognosis in severely injured patients
Source: Sci Rep. 2021 May 26;11:11031. doi: 10.1038/s41598-021-90492-0 (PMC8154895; doi:10.1038/s41598-021-90492-0)
Supplement: Supplementary file 2 — Supplementary Legends. [file 41598_2021_90492_MOESM2_ESM.docx]

Supplementary Figure 1. Flow diagram showing the patient screening and enrolment.
